# Extracorporeal Therapies in Children with Acute Liver Failure: A Single-Center Experience

**DOI:** 10.5152/tjg.2022.22062

**Published:** 2023-01-01

**Authors:** Emrah Gün, Ayşen Durak, Edin Botan, Selen Şimşek Pervane, Anar Gurbanov, Burak Balaban, Fevzi Kahveci, Hasan Özen, Hacer Uçmak, Fulden Aycan, Zarife Kuloğlu, Tanıl Kendirli

**Affiliations:** 1 Department of Pediatric Intensive Care, Ankara University Faculty of Medicine, Ankara, Turkey; 2 Department of Pediatric, Ankara University Faculty of Medicine, Ankara, Turkey; 3 Department of Pediatric Gastroenterology, Ankara University Faculty of Medicine, Ankara, Turkey

**Keywords:** Children, continuous renal replacement therapy, liver failure, pediatric intensive care unit, therapeutic plasma exchange

## Abstract

**Background:**

The aim of this study is to determine the indication, timing, and administration of extracorporeal therapies such as total plasma exchange and continuous renal replacement therapy in children with acute liver failure or acute-on-chronic liver failure.

**Methods:**

This study is conducted as a retrospective, single-center study. Between January 2016 and December 2021, pediatric acute liver failure or acute-on-chronic liver failure patients for whom total plasma exchange and/or continuous renal replacement therapy was performed were included in this study.

**Results:**

Thirty-four children with acute liver failure or acute-on-chronic liver failure were included during the study period. The children comprised 14 (41.1%) males, and the median age of the patients was 54 months (5-21). Twenty-four patients (70.6%) had pediatric acute liver failure, and 10 patients (29.4%) had acute-on-chronic liver failure. Patients’ median model for end-stage liver disease and pediatric end-stage liver disease scores were 24.7/23.5, respectively. Total plasma exchange therapy was performed on all patients whereas continuous renal replacement therapy was performed on 13 patients (38.2%). The median duration of continuous renal replacement therapy was 2.5 days (2-24). The median number of the total plasma exchange sessions was 3 (1-20). The median length of stay in pediatric intensive care unit was 4.5 (2-74) days. Eleven (32.5%) patients had 1 or more improvements in hepatic encephalopathy scores after extracorporeal therapy. Eleven (32.5%) patients died. There was a significant difference between the survivors and non-survivors with respect to levels of albumin, ammonia, pediatric risk of mortality scores, and pre-hepatic encephalopathy scores. Liver transplantation was performed in 4 of 24 pediatric acute liver failure patients, and all of them survived.

**Conclusion:**

Total plasma exchange and continuous renal replacement therapy are life-saving, and both methods may reduce morbidity and mortality, also bridging to liver transplantation.

## Introduction

Pediatric acute liver failure (pALF) is a severe clinical state, and it can cause encephalopathy, multiorgan failure (MOF), shock, and death in short time.^1,2^ Hepatocyte necrosis is followed by the release of cytokines and adhesion molecules resulting in proinflammatory cascade.^2^ Although rare in the pediatric population, it is associated with a high mortality rate.^3,4^ The pALF comprised 10%-15% of all pediatric liver transplants.^4^ The transplant-free mortality rate has been reported as 55%.^5^ Acute-on-chronic hepatic failure (ACLF) is defined as acute decompensation in a patient with chronic liver disease (CLD). Although it is more common in clinical practice, there are little data on intensive care management, especially in children.^6^ The mortality rate of pediatric ACLF ranged between 20% and 30%.^7^ Compared to CLD, the mortality of pediatric ACLF was 5 times higher.^7^ Considering the general scarcity of organ donation, transplant lists lead to long waiting times between donors and recipients, and the management options are critical in terms of survival during this waiting period. The chart management for pALF and ACLF aims to stabilize vital organ functions, removes circulating cytokines and toxic metabolites, and provides deficient plasma factors. The released cytokines are primarily responsible for the progression of MOF.

Extracorporeal therapies reduce ammonia and maintain fluid balance and cytokine homeostasis. The most common extracorporeal therapies are continuous renal replacement therapy (CRRT) and therapeutic plasma exchange (TPE).^8^ The other extracorporeal therapies include extracorporeal membrane oxygenation (ECMO) and molecular adsorbent recirculating system (MARS).^9,10^ In critically ill children with pALF and pediatric ACLF, CRRT, TPE, and MARS for the removal of these toxins have attracted attention recently.^8,11^ Continuous renal replacement therapy can be used in the treatment of acute renal failure, hyperammonemia (>150 mmol/L), and fluid overload accompanying MOF.^12^ Therapeutic plasma exchange can be used in the management of coagulopathy.^10,13,14^ This study aimed to analyze the indication, timing, and administration of extracorporeal therapies such as TPE and CRRT in children with ALF or ACLF.

## MATERIALS AND METHODS

This study was conducted as a retrospective, single-center study. Between January 2016 and December 2021, 34 pALF patients for whom TPE or TPE\CRRT was performed were included in this study. Patients also received conventional hepatic failure and hepatic encephalopathy (HE) medical treatment options. The approval was obtained from the Institutional Review Board (Ankara University Faculty of Medicine) (Approval Number: İ11-717-21).

For all of them, a central venous catheter was used as a dialysis catheter for TPE or/and CRRT. Firstly, the internal jugular vein was preferred for temporary double-lumen hemodialysis catheter, and the catheter size varied between 7F and 12.5F according to the patient’s weight.

Patients were identified from the institutional database based on demographic data, the number of sessions received in CRRT and TPE, baseline clinical and biological patient’s values, HE and other outcomes, mortality, and length of stay (LOS) in pediatric intensive care unit (PICU). Pre and post-therapy HE, Model for End-Stage Liver Disease (MELD) and Pediatric End-Stage Liver Disease (PELD, for under 12 years old) scores were determined. Model for End-Stage Liver Disease and PELD scores at www.mdcalc.com or “Organ Procurement and Transplantation Network” (OPTN; www. optn.transplant.hrsa.gov) were automatically calculated from websites. HE was scored according to West Haven criteria or assesed with age-based modified encephalopathy rating scores.^1^

Setup and troubleshooting during TPE sessions were performed bu apheresis nurses. Follow up of the CRRT process was performed by PICU nurses. Patients were also evaluated bu Pediatric nephrology, gastroenterology and transplantation transplantation teams. Continuous renal replacement therapy indications were defined as oliguric acute kidney injury, fluid overload, and hyperammonemia. The fluid overload (FO) for each child was determined at the time of CRRT initiation using the intensive care unit (ICU) admission weight in kilogram as the baseline weight of comparison as shown below. (Using the method described by Goldstein et al.)^15^

A Prismaflex (Baxter, US) device was used for CRRT in all patients. During the treatment, the blood flow rate was set as 4-12 mL/kg/min. Dialysate flow rates were set between 2000 and 5000 mL/m^2^/1.73 and replacement fluid flow rate was 2000 mL/m^2^/1.73. As CRRT circuit, HF20, M60, and M100 membranes were used during RRT.

Therapeutic plasma exchange procedures were performed using the Spectra Optia Centrifugal Apheresis System (Terumo BCT Inc., USA). All of the patients received high-volume plasma exchange (HVP). The fresh frozen plasma was preferred as the replacement fluid, and acid citrate dextrose was used for anticoagulation, and calcium gluconate supplementation was used if calcium ion was <1.0. TPE-HV category I and grade 1A ALF. Daily TPE was performed liver transplantation (LT) or self-regeneration occurs.^16^ We performed high-volume (1.5 approximately plasma volumes) and centrifugation-based apheresis.

Pediatric ALF was defined using the following criteria: “(1) no known history of CLD; (2) biochemical evidence of ALF (raised transaminases) <onset 8 weeks; (3) hepatic-based coagulopathy defined as a prothrombin time (PT) ≥15 seconds or international normalized ratio (INR) ≥1.5 not corrected by vitamin K in the presence of clinical HE or a PT ≥20 seconds or INR ≥2.0 regardless of HE.”^17^

Pediatric ACLF was defined using the following criteria: an acute hepatic insult manifesting as jaundice (serum bilirubin >5 mg/dL) and coagulopathy (INR >1.5) complicated within 4 weeks by clinical ascite and/or HE in a patient who had CLD previously.^18,19^

### Statistical Analysis

Statistical analysis was done with the Statistical Package for Social Sciences version 26.0 (IBM Corp.; Armonk, NY, USA). Continuous data are reported as median (interquartile range [IQR] 25-75 percentile), and categorical data are reported as percentages. Mann–Whitney U, Wilcoxon rank-sum test, and Fisher’s exact test were used for comparisons as appropriate. Probability estimates for survival were made with Kaplan–Meier survival analysis. If *P*-value was lower than .05, it was considered statistically significant.

## Results

Thirty-four children with ALF were included during the study period. The children comprised 14 (41.1%) males, and the median age of the patients was 54 months (5-211). Twenty-four patients (70.6%) had ALF, and 10 patients (29.4%) had ACLF. Among the patients with ALF, the common etiology was toxic hepatitis in 17, followed by metabolic disease in 2, infections in 4, Wilson disease in 2, idiopathic in 3, and heart failure in 2. The toxic agent was determined as acetominophen in 3, inhaled anesthetic in 2, mushrooms in 6, and antibiotics in 5 and others (Table 1).

Patients’ median MELD and PELD scores were 24.7 (0.9-40.6) and 23.5 (6.0-40.0), respectively. Total plasma exchange therapy was performed on all patients whereas CRRT was performed on 13 patients (38.2%). Continuous renal replacement therapy was performed in 5 patients due to removal of the toxic substance. Other CRRT indications and TPE indications are given in Table 1. The median duration of CRRT was 2.5 days (2-24). The median number of the TPE sessions was 3 (1-20). We performed invasive mechanical ventilation in 14 patients (41.1%), and the median LOS in PICU was 4.5 (2-74) days. Patients were generally well on extracorporeal treatments and tolerated and completed them as prescribed. Total plasma exchange complications developed during the procedure in 10 patients (29.4%), hypocalcemia developed in 5 patients, hypotension in 3 patients, and anemia in 4 patients.

Eleven (32.5%) patients had 1 or more improvements in HE scores after extracorporeal therapy. Ten of 23 survivor patients had at least 1 grade of improvement in HE scores, and only 1 patient with improvement in HE scores died.

**Figure 1.**
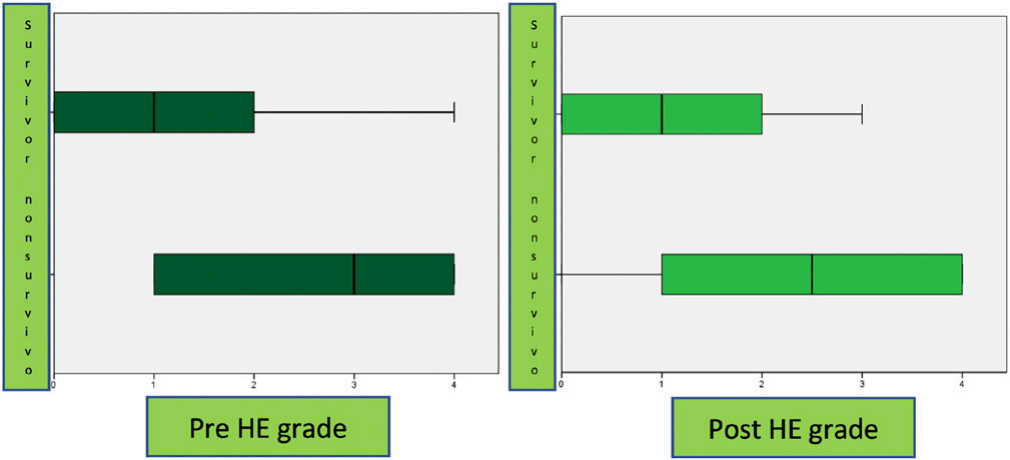
Comparison of HE grade before and after PEX in survivors and non-survivors group. HE, hepatic encephalopathy.

Eleven (32.5%) patients died. There was a significant difference between the survivors and non-survivors with respect to levels of albumin, ammonia, and Pediatric Risk of Mortality (PRISM) scores (Table 2). There was no significant difference between the survivors and non-survivors with respect to PELD scores, MELD scores, CRRT number of days, TPE number of days, and LOS in PICU. While there was no significant difference between post-HE scores, there was a significant difference between pre-HE scores (Figure 1). Liver transplantation was performed in 4 of 24 pALF patients, and all of them survived. Three patients (8.8%) received ECMO support and died.

**Figure 2.**
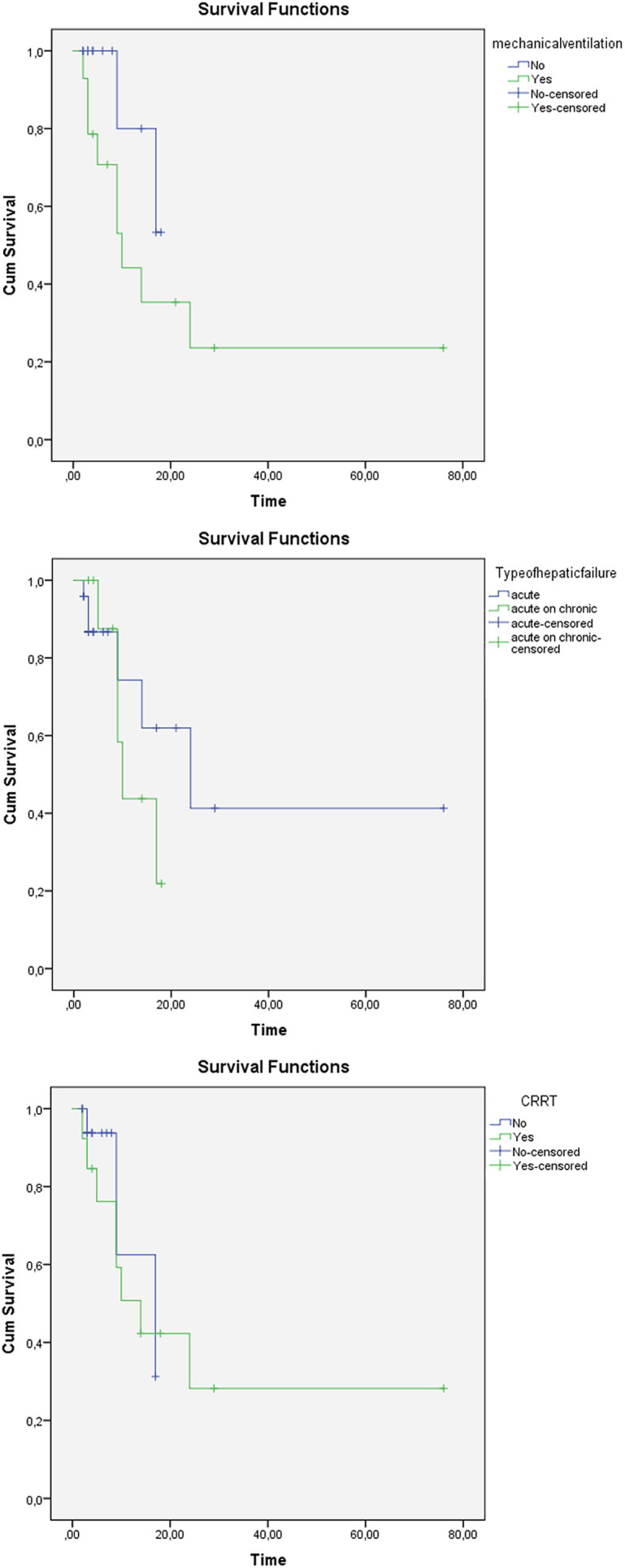
Kaplan–Meier survival curves for mechanical ventilation support, hepatic failure type, and CRRT therapy. CRRT, continue renal replacement therapy.

The survival time analysis was calculated by Kaplan–Meier method. The survival time showed no significant differences in CRRT therapy and hepatic failure type (*P* = .36 and *P* = .86, respectively) but showed a significant difference in mechanical ventilation support (*P* = .026). These data are given in Figure 2.

## Discussion

Despite recent developments, pALF remains a rare disease with high mortality rate. In some studies, its recovery without liver transplantation has been reported as 48%.^20^ In our study, this rate was 70%. In previous studies, the frequency of liver transplantation for ALF ranged from 21% to 41%.^20^ In our study, this rate was 16.6%. Unlike Western countries, pediatric liver transplantation, mostly liver graft donation from a living donor, is performed in our country. Acute liver failure and ACLF is a devastating complex pathophysiological process that can result in rapid death with MOF.^10,21^ The primary goal of an ideal treatment should be to stop the cascade that leads to this devastating process, to remove circulating blood.^10^ In addition, acute kidney injury complicates LF, and patients receiving high volumes of drugs and blood product transfusion are at risk of fluid overload.^10^ Due to this nature of the disease, plasma products used for the correction of coagulopathy increase the protein load and aggravate hyperammonemia.^10^ Therefore, using TPE and CRRT in combination makes sense and can provide a successful bridge to liver transplantation.^8,10,22^

Plasmapheresis removes plasma cytokines and drivers of systemic inflammatory cascade. It is an extracorporeal treatment method used in acute liver failure.^21^ High-volume plasma exchange is recommended as category I and grade 1A in the management of acute liver failure in the American Society for Apheresis (ASFA) guideline.^16^ In our study, the median number of the TPE sessions was 3 (1-20) This was similar to previous studies.^20,23,24^

Ammonia plays a central role in the pathogenesis of central nervous system toxicity in LF, and high ammonia levels have been reported as a poor prognostic factor for herniation and cerebral edema.^25^ However, there is still no consensus on a threshold ammonia value for initiating extracorporeal therapy. Ammonia thresholds for CRRT initiation and dose of treatment have both been a matter of debate.^26^ In our study, the mean ammonia values before extracorporeal treatment was found to be 105.5 (28.0-358.0) μmol/L. There was a significant difference between the baseline ammonia values of the non-survivor and the survivors (153.0 (107.0-358.0) vs 82.0 (28.0-290.0), *P* = .008).

Survival of patients with hepatic failure requiring the addition of CRRT to clinical therapy was at the rate of 30%-35%.^27^ Improved survival of 54%-58% has been reported recently.^28^ In our study, the mortality rate was determined as 32.3%. We reported regression in encephalopathy scores after CRRT/TPE or TPE in 11 patients (32.5%). There was no significant difference post-HE scores between survivors and non-survivors (*P* = .104).

There was no significant difference between non-survivor and survivor patients in terms of the scales used to calculate the severity of liver disease (PELD and MELD scores), INR, total/conjugated bilirubin, alanine aminotransferase and aspartate aminotransferase value. Similar results were obtained in a study by Akcan Arikan et al^29^ which examined a cohort of 15 patients. As a result, TPE/CRRT bridges transplantation or spontaneous recovery. It approves that with scoring systems, it is not possible to predict who will survive and that it is difficult to determine a value to start extracorporeal therapy.

We were successfully able to use TPE and TPE/CRRT. No treatment-related complications developed. Median CRRT duration days were set as 2.5 (2-24) and TPE sessions were set as 3 (1-20). In another study by Akcan Arıkan et al. CRRT application time was determined 19 (5-50) days and the number of TPE seans was determined 4 (1-8). Another study by Schafer et al reported CRRT/TPE time as 5.7 (4.5-6.6) hours. Treatments in pALF/ACLF are significantly heterogenous, and there are non-standardized treatment protocols. Also, sampling durations for biomarkers are not standardized, one particular consideration is important for intermittent treatments.^11,30^ Survivors and non-survivors had similar PICU length of stay. Survivors and non-survivors had similar PICU length of stay (*P* = .344). Akcan Arıkan et al reported that non-survivors had shorter LOS in PICU.

Liver support devices have been shown to improve biochemical parameters, neurological status, and hemodynamical stability.^2,20-22^ In our study, similar to these studies, improvement in neurological status and hemodynamical stability was shown.

As with HVP, albumin-based dialysis aims to remove albumin-bound toxins and water-soluble substances. Albumin-based dialysis appears to have some of the same clinical effects as HVP, such as improving systemic vascular resistance, blood pressure, and HE grade in adults.^31^ Albumin-based dialysis (MARS, prometheus) was performed only in selected centers with experience, although there is no evidence of survival seen in adults or children.^22^ Albumin-based dialysis cannot be performed in our center.

Our study had a few limitations. This study has small sample size, and it was conducted at a single center and also as a retrospective study.

## Conclusion

Pediatric ALF and ACLF are critical diseases that can progress rapidly, and they are associated with severe morbidity and mortality, but this clinical situation is better resulted with well-managed medical therapies and extracorporeal support. Especially, TPE and CRRT are very important in this devastating situation. Both methods are available in PICUs and they are life-saving and can reduce morbidity and mortality, also bridging to liver transplantation.

## Figures and Tables

**Table 1:** Demographics and Baseline, Clinical, and Biological Patient’s Values

	Median (Min-Max) or n (%)
Age (month)	54 (5-211)
Male	14 (41.1%)
**TPE indication**	
Elevated coagulation markers	31
Toxic hepatitis	4
TAMOF	2
Acute-on-chronic liver failure	10 (29.4%)
International normalized ratio	2.8 (1.05-17.2)
Total bilirubin, mg/dL	6.1 (0.57-58.7)
Conjugated bilirubin, mg/dL	2.6 (0,.39-33.8)
Albumin, g/dL	3.1 (2.2-4.2)
Ammonia at TPE-CRRT start, μmol/L	105.5 (28.0-358.0)
Alanine aminotransferase	694.5 (19-8407)
Aspartate aminotransferase	907 (68-9665)
Pediatric end-stage liver disease/model for end-stage liver disease	24.7 (0.9-40.6)/23.5 (6.0-40.0)
PRISM	12.0 (2-48)
Acute kidney injury*	12 (35.2%)
Invasive mechanical ventilation	14 (41.1%)
Continuous renal replacement therapy, n	13 (38.2%)
Continuous renal replacement therapy duration, days	2.5 (2-24)
**CRRT indication**	
Fluid overload	8
Acute kidney injury	7
Toxic substance removal	5
Hyperammonemia	13
Mortality, n	11 (32.3%)
TPE, days, median (IQR 25-75)	3 (1-20)
PICU duration, days, median (IQR 25-75)	4.5 (2-74)
Pre-hepatic encephalopathy score, median (IQR 25-75)	1.0 (0-4.0)
Any improvement in HE scores after PEX or CRRT, n (%)	11 (32.5%)

*Acute kidney injury was assessed at the time of continuous renal replacement therapy start. Values are expressed as number (%) or median value (range).

PICU, pediatric intensive care unit; CRRT, continue renal replacement therapy; TPE, total plasma exchange; PRISM, pediatric risk of mortality scores; TAMOF, thrombocytopenia-associated multiple-organ failure.

**Table 2.** Effect of Extracorporeal Therapy as a Bridge to Transplant and Overall Survival

	Survivors (n = 23)	Non-survivors (n = 11)	*P*
pALF	18	6	.232*
Pediatric ACLF	5	5	.232*
International normalized ratio	2.9 (1.05-17.2)	2.0 (1.31-7.9)	.637
Total bilirubin, mg/dL	3.9 (0.57-43.4)	11.1 (2.09-58.7)	.065
Conjugated bilirubin, mg/dL	2.4 (0.39-26.2)	6.1 (0.89-33.8)	.091
Albumin, g/dL	3.37 (2.6-4.2)	2.6 (2.2-3.8)	**.004**
Ammonia at extracorporeal therapy start, μmol/L	82.0 (28.0-290.0)	153.0 (107.0-358.0)	**.008**
Alanine aminotransferase	927.0 (33.0-8407.0)	245.0 (19.0-2528.0)	.201
Aspartate aminotransferase	952.0 (73.0-9733.0)	756.0 (68.0-3996.0)	.445
Serum creatinine	0.35 (0.24-0.47)	0.68(0.585-0.86)	.133
Sodium	138 (135-141)	152 (144-153)	.326
Potassium	3.8 (3.5-4.1)	3.4 (3.2-3.7)	.228
White blood cell	8050 (5245-10 565)	20 040 (11 760-22 920)	**.004**
Platelets	165 000 (10 6000-28 7000)	55 000 (42 500-69 000)	**.034**
Hemoglobin	10.9 (8-11.9)	9.4 (8.6-12.35)	.176
PT	33.8		.537
Lactate	2.7 (2.0-3.0)	5.6 (5.05-6.95)	**.002**
pH	7.43 (7.40-7.47)	7.54 (7.45-7.58)	.736
BUN	11 (6-16)	19 (16.5-24.2)	.014
Pediatric end-stage liver disease/model for end-stage liver disease	21.4 (0.9-40.6) 20.0 (6.0-32.0)	31.0 (16.8-36.3) 28.0 (27.0-40.0)	.082 .067
PRISM	8.0 (2-31)	31.0 (8-48)	**.001**
CRRT, days	2.0 (2.0-10.0)	5.5 (2.0-24.0)	.283
TPE, days	3.0 (1.0-20.0)	4.0 (1.0-9.0)	.913
LOS in PICU, days	4.0 (2.0-76.0)	9.0 (2.0-24.0)	.344
Pre-HE scores	1.0 (0.0-4.0)	3.0 (0.0-4.0)	**.019**
Post-HE scores	1.0 (0.0-3.0)	2.5 (0.0-4.0)	.104

HE, hepatic encephalopathy; PRISM, pediatric risk of mortality scores; PICU, pediatric intensive care unit; CRRT, continue renal replacement therapy; TPE, total plasma exchange; pALF, pediatric acute liver failure; ACLF, acute-on-chronic hepatic failure.*P*-values lower than .05 were considered statistically significant.
